# Effects of preemptive analgesia with intravenous acetaminophen on postoperative pain relief in patients undergoing third molar surgery: a prospective, single-blind, randomized controlled trial

**DOI:** 10.4317/medoral.23983

**Published:** 2020-10-09

**Authors:** Keita Kano, Kahori Kawamura, Tatsuro Miyake

**Affiliations:** 1 Graduate School of Dentistry, Osaka Dental University, Osaka, Japan; 2 Department of Preventive and Community Dentistry, Osaka Dental University, Osaka, Japan

## Abstract

**Background:**

The efficacy of preemptive analgesia in managing postoperative pain remains controversial. The aim of this study was to compare the efficacy of intravenous (IV) acetaminophen administered before or immediately after the surgical extraction of an impacted mandibular third molar.

**Material and Methods:**

This prospective randomized clinical trial included 120 patients. The patients were assigned to one of three groups: the preoperative-treatment group (pre-group), which received 1000 mg of IV acetaminophen 20 min before surgery; the postoperative-treatment group (post-group), which received 1000 mg of IV acetaminophen after surgery; the no-treatment group (control-group), which did not receive any analgesic. Rescue analgesic (60 mg loxoprofen) was issued to each patient, with instructions on self-administration if needed. For the rescue medication usage, the time of first loxoprofen usage and the total amount of loxoprofen consumption were obtained for a 17-hour period after surgery. We measured pain using the visual analogue scale at 1 hour and at 2, 3, 4, 5, and 15 hours after surgery.

**Results:**

There was no significant difference in pain level among the three groups at any time interval. However, the pre-group demonstrated significantly lower rescue analgesic consumption and longer time until initial administration.

**Conclusions:**

Administration of IV acetaminophen before third molar surgery provides more effective pain control than postoperative administration and no treatment.

** Key words:**Preemptive analgesia, acetaminophen, impacted third molar, pain relief, randomized controlled trial.

## Introduction

The extraction of wisdom teeth, or third molars, is the most common procedure in the field of oral and maxillofacial surgery. The removal of an impacted lower third molar is particularly invasive and often associated with postoperative pain, swelling, and trismus, which frustrates patients (1). Effective pain treatment is an important component of healing. In the conventional management of postoperative pain, patients self-administer analgesics in response to perceptions of pain. However, once severe pain occurs, it can be difficult to manage successfully with analgesics. Given the predictability of third molar surgical pain, a preventive approach to postoperative pain management may yield better results.

Preemptive analgesia is one of the many strategies for pain management. This approach involves managing pain before its onset to minimize postoperative pain by interrupting afferent input. Thus, the most effective preemptive agents for reducing central sensitization are analgesics that act on pain due to injuries caused by incisions and the associated inflammation (2).

The efficacy of preemptive analgesia in managing postoperative pain remains controversial. Previous randomized control trials have concluded that preemptive analgesia improves postoperative pain (3,4). However, other studies have concluded that postoperative administration of analgesia is more effective in improving postoperative pain (5,6) or that there is no difference between preemptive and postoperative administration (7). Variations in experimental interventions, methods, terminology, and definitions have resulted in conflicting research findings (8). Further research on this subject is required due to the scarcity of such studies in the oral surgery literature.

Acetaminophen has both central and peripheral effects, similar to nonsteroidal anti-inflammatory drugs (NSAIDs). Acetaminophen acts to inhibit the synthesis of prostaglandin (9) and block pain mechanisms in the spinal cord. Acetaminophen is a safe and effective analgesic that can cross the blood-brain barrier, which allows for accumulation of high concentrations of the drug in the cerebrospinal fluid. Additionally, it is known to act as an antinociceptive (10).

To the best of our knowledge, this is the first study investigating preemptive intravenous (IV) acetaminophen as a method to reduce postoperative pain and evaluating the use of rescue analgesics following lower third molar surgery. The aim of this study was to compare the efficacy of rescue IV acetaminophen administered before or immediately after the surgical extraction of an impacted mandibular third molar in three groups of patients: those who received preemptive acetaminophen, postoperative acetaminophen, or no analgesic.

Our study was designed to evaluate preoperative IV acetaminophen in a prospective, single-blind, randomized, clinical trial. Our hypothesis was that preemptive acetaminophen is superior to either acetaminophen used postoperatively or no analgesic. Our outcome was patient-reported postoperative pain and the usage of rescue analgesics used by patients following lower third molar surgery.

## Material and Methods

- Patients

This randomized, single-blind study was performed on patients undergoing single mandibular third molar surgery between September 2017 and May 2019 in the Department of Dentistry and Oral Surgery at the Uji Takeda Hospital. A total of 120 patients between 20 and 68 years old were enrolled according to the Consolidated Standards of Reporting Trials (CONSORT) protocol (11). A copy of the subject matter, aims, and risks of the study was provided to all patients, and they all provided written consent to participate. The inclusion criteria for this study were a minimum age of 20 years; an American Society of Anesthesiologists (ASA) physical status of 1; planned extraction of a third molar classified as IIB, IIIB, IIC, or IIIC (according to the Pell and Gregory classification system) in an inpatient setting under IV sedation; and agreement to follow the study protocol. The exclusion criteria were pregnancy or suspicion of pregnancy, allergy and/or contraindication to acetaminophen, history of alcohol or drug abuse, either receiving any anti-inflammatory/pain medications or having a chronic pain condition at the time of the study, and active infection of the third molars with pus, edema, and trismus. The participants were randomly assigned to one of the following groups: a preoperative-treatment group (pre-group), which received 1000 mg of IV acetaminophen 20 min before surgery; a postoperative-treatment group (post-group), which received 1000 mg of IV acetaminophen after surgery, and a no-treatment group (no-group), which acted as control. Rescue analgesic (60 mg of loxoprofen) was issued to each patient, with instructions to take the medication if deemed necessary.

Randomization was based on the opaque, sealed-envelope technique. A total of 120 identical opaque sealed envelopes labeled with a serial number, each containing the name of one of the groups (with 40 envelopes per group), which was determined in advance by the function “randbetween” in Microsoft Excel version 2016 (Microsoft, Redmond, WA, USA), were prepared before the initiation of the study. The resident dentist, who was not involved in patient care, selected an envelope and directed the pharmacy to prepare the analgesia medications. This trial was single-blind; i.e., only patients were blinded to the treatment groups.

- Surgical procedure

All surgeries for this study were performed by a single surgeon-assistant pair. After confirming the patient’s fasting status, IV access was established using a 22-gauge Intracath (Terumo, Tokyo, Japan) catheter. Routine noninvasive blood pressure, heart rate, and peripheral oxygen saturation monitors were connected to the patient. Oxygen was delivered via nasal cannula at 1 L/min. Sedation medication and local anesthesia were administered by the surgeon prior to the procedure. Sedation was performed using 0.075 mg/kg of midazolam as a single IV bolus. Local anesthesia was performed by blocking the inferior alveolar, lingual, and buccal nerves with two 1.8-mL capsules of 2% lidocaine with 1:80,000 epinephrine (Dentsply Sirona, Tokyo, Japan). To standardize the resulting surgical trauma, the surgery was performed using the classic full-flap technique, with bone removal and tooth sectioning based on the condition of the third molar. Suturing was performed using 4-0 silk. The duration of the operation (from first incision to final suture) was recorded.

- Outcome variables

The primary outcome variable was self-reported postoperative pain. Patients were instructed to complete a report every hour for the first 5 to 15 hours after surgery. We used the visual analogue scale (VAS) for assessment of postoperative pain, which ranges from “no pain” to “pain that could not be more severe.” Patients were instructed to mark a point between these extremes that represented their pain levels. The secondary outcome variable was the time at which the initial dose of the rescue medication was taken and the subsequent frequency of administration. Additionally, patients were instructed to record the number of pills taken and the time of analgesia use for 17 hours after surgery. We collected adverse events data to evaluate the safety of the analgesic regimen.

- Statistical methods

Using G*Power version 3.0.10 for Windows (Franz Faul, Kiel University, Germany), it was determined that 34 patients per group were needed to achieve 80% power with 95% confidence. Forty patients were enrolled in each group to account for possible dropouts. Statistical analysis was performed using SPSS version 26 for Windows (IBM, Armonk, NY, USA) and the R package version 3.4.1 for Windows (The R Foundation for Statistical Computing, Vienna, Austria). A *p* value <0.05 was considered statistically significant for all analyses. Data are presented as mean values and standard deviation. The demographic data of the study groups were compared using the chi-squared (χ2) test for qualitative variables and one-way analysis of variance for quantitative variables. Differences in variables, such as surgery duration, VAS pain scores at each of the fixed time intervals, the time until the initial intake of rescue analgesic, and total analgesic consumption between groups, were analyzed using the Kruskal-Wallis test. If the Kruskal-Wallis test indicated a statistically significant difference between groups for any given variable, the Mann-Whitney U test with Bonferroni correction was applied to identify the group that demonstrated the statistical difference. The proportion of subjects requiring the rescue medicine was compared between the study groups using the log-rank test. Cox proportional hazard models were used to further analyze the results.

## Results

- Demographic and clinical data

**Figure 1:**
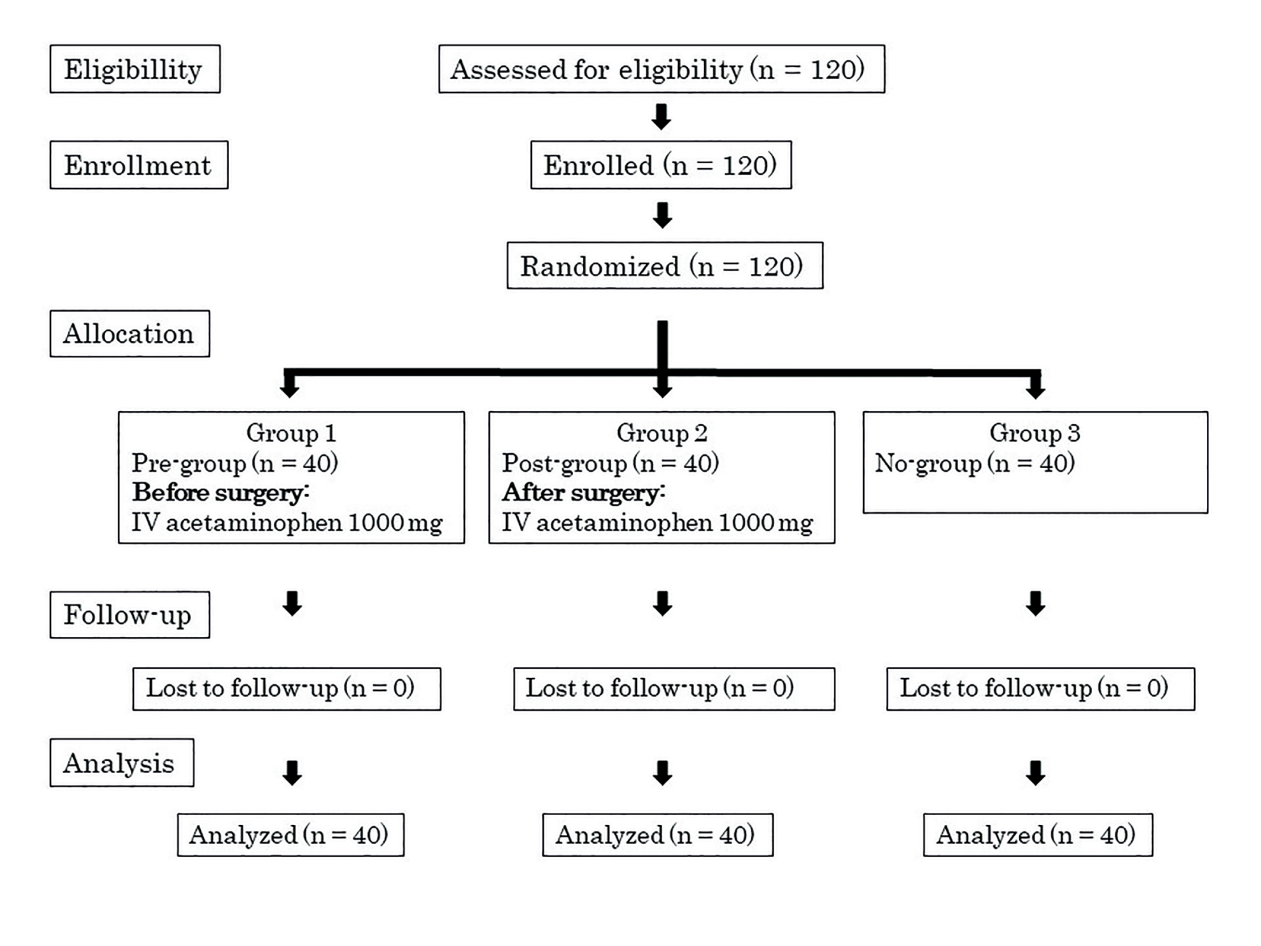
Flowchart of patient recruitment and study group randomization, according to the CONSORT statement. Pre-group, acetaminophen before surgery; Post-group, acetaminophen after surgery; No-group, control.

A total of 120 patients were recruited to participate in this study, and 120 patients (38 men and 82 women) between 20 and 68 years old (32.5 ± 11.9 years) completed the study and filled out all required reports. No data were missing. A flowchart of patient participation in this study is shown in Fig. 1. There were no significant differences in sex, age, height, weight, operation duration, or difficulty of surgery among the three groups (Table 1).

**Table 1:**
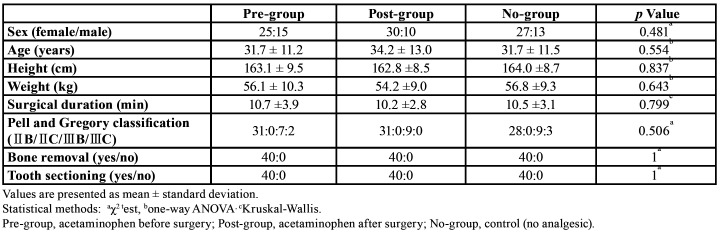
Demographic data.

- Analysis of VAS

The pre-group and the post-group exhibited peak pain elevation 2 hours after the surgical procedure (3.30 ± 2.80 and 2.74 ± 1.93 hours, respectively), and the no-group exhibited an elevated pain peak 3 hours after the surgical procedure (3.38 ± 2.14 hours). There was no significant difference in pain level at each fixed time interval among the three groups (Table 2).

- Time of initial intake of rescue medication

There were significant differences among the groups with regard to the timing of initial rescue analgesic intake (*p* = 0.001). Further analysis revealed significant differences between the pre-group and the no-group (*p* = 0.003) and between the post-group and the no-group (*p* = 0.016). There was no significant difference between the pre-group and the post-group (*p* = 0.774) (Table 3).

**Figure 2:**
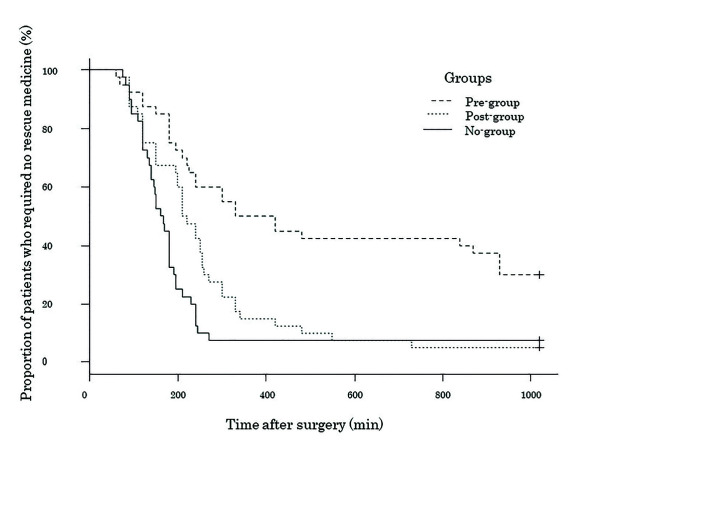
Kaplan–Meier plot for the three groups, representing the proportion of patients in each group who did not require rescue medicine (χ2 = 23.9, df = 2, *p* < 0.001, log-rank). Pre-group, acetaminophen before surgery; Post-group, acetaminophen after surgery; No-group, control.

In addition, the proportion of subjects not requiring rescue analgesic was significantly higher in the pre-group throughout the observation period (Fig. 2). Further analysis revealed that the probability of a patient from the pre-group needing to take rescue analgesic was 70% lower than that of a patient from the no-group (Table 4).

- Amount of rescue analgesic consumption

There were significant differences in total rescue analgesic consumption (*p* = 0.01). Further analysis revealed a significant difference between the pre-group and the no-group (*p* = 0.014). There were no significant differences between the pre-group and the post-group (*p* = 0.256) or between the post-group and the no-group (*p* = 0.333) (Table 3).

- Adverse events

There were no complications associated with the surgical procedure. There were no serious adverse events, such as nausea or vomiting, due to pharmacological interventions in any of the groups.

**Table 2:**
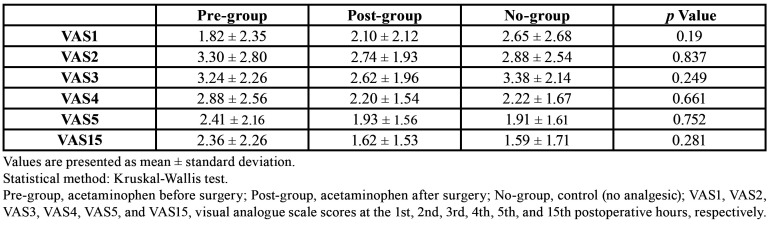
Pain intensity on the visual analogue scale.

**Table 3:**
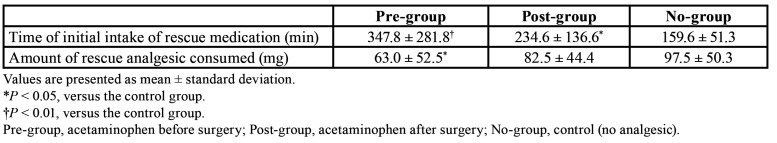
Objective measurement data.

**Table 4:**
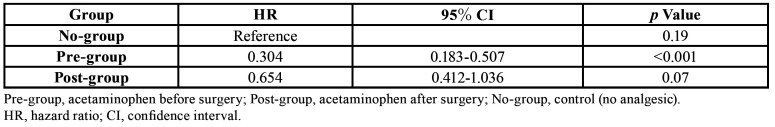
Cox proportional hazard analysis of the proportions of patients who did not require rescue medicine.

## Discussion

Several clinical trials have examined different approaches to minimize pain and curtail the need for additional analgesics to reduce the incidence of adverse effects following third molar extractions. Preemptive analgesia is one such approach and has been studied extensively. A relationship exists between operative tissue damage and intense postoperative pain, both acute and chronic, as a result of tissue damage, which activates nociceptors (12). The preemptive analgesia approach in which intervention precedes incision offers a more effective means of controlling postoperative pain and preventing central sensitization and chronic neuropathic pain than identical interventions applied after the incision (8).

Previous studies that have attempted to assess the efficacy of preemptive analgesia in patients undergoing oral surgery used protocols in which the outcome of preoperative administration was only compared against outcomes of either preoperative placebo administration (13) or postoperative analgesic administration (14). Such designs could be the reason for the lack of consensus regarding the comparative efficacy of preemptive analgesic interventions against the conventional postoperative approach for managing acute pain. According to Kaczmarzyk *et al*. (15), studies require a comparison of preoperative and postoperative outcomes to demonstrate preemptive analgesic effect. This study was designed using the common standard for clinical investigations on preemptive analgesia and draws comparisons among the following treatment groups: preoperative administration, postoperative administration, and no treatment. To standardize operative and postoperative environments, all patients underwent a single molar surgery for the same classification of molar impaction and were hospitalized for 1 day.

Previous clinical trials on preemptive analgesia typically used NSAIDs as analgesics (1). No previous study has examined the effect of preemptive IV acetaminophen on postoperative pain and rescue analgesic use in patients who have undergone lower third molar surgery. Theoretically, acetaminophen acts within the spinal cord by blocking mechanisms that, if uninterrupted, may enhance central sensitization. The benefits of acetaminophen include easy passage through the blood–brain barrier, an ability to reach high concentrations in the cerebrospinal fluid, and an antinociceptive effect, which ensure its effectiveness as an analgesic (10). Furthermore, acetaminophen has been approved for use in the treatment of acute pain and fever in adults and children older than 2 years by the US Food and Drug Administration since 2010 (16). In Japan, we have been able to use IV acetaminophen with an increase in maximum prescribing dose from 1500 to 4000 mg/day, which was approved in November 2013.

In this study, we found no differences in treatment outcomes among patients who received preemptive acetaminophen analgesia, postoperative acetaminophen analgesia, and no treatment, as measured by the VAS. Ethically, all analgesic evaluations must ensure patient protection; thus, all our patients had access to rescue medication for use according to their pain scale. All patients reported sTable postoperative pain, resulting in no differences in the VAS scores among the patients. Our aim was to assess the effect of a single IV administration of acetaminophen. Patients could take rescue medicine to address their perception of pain after surgery. If rescue medicine intake was limited, different results may have been attained. Moreover, Ong *et al*. (17) suggest that preemptive analgesia is better assessed by measuring the time between the end of surgery and starting analgesics as well as the total analgesic dosage rather than by measuring the perception of pain.

The first 12 hours after surgical extraction of an impacted lower third molar are considered to be the worst in terms of perceived pain (18). If there are no inflammatory complications, pain is usually absent or negligible after the second postoperative day (15). One of the goals of preemptive analgesia is the prevention of pain sensation during this peak pain period (15). In this study, patients receiving preoperative acetaminophen waited a longer period before taking rescue medicine (median time: 375 min) than patients in the postoperative acetaminophen and control groups, which had median times of 215 and 163 min, respectively. Furthermore, only 70% of the patients who received preoperative acetaminophen required at least one dose of rescue medicine, compared with 95% of patients who received postoperative acetaminophen and 92.5% of patients in the control group. We believe that preemptively delivering acetaminophen can prevent central sensitization, causing its analgesic effect to last longer.

Studies that have investigated the use of preemptive acetaminophen during other surgeries have demonstrated similar results. Koteswara *et al*. (19) recruited 39 patients undergoing functional endoscopic sinus surgeries and allocated them to one of two groups, one in which IV paracetamol was administered before surgery and another in which IV paracetamol was administered after surgery. Pain intensity (using the VAS), time of initial analgesic use, and total analgesic consumption were then compared. The group that received preoperative IV paracetamol demonstrated a significantly lower VAS pain score, lower rescue analgesic consumption, and later initial use of the rescue analgesic than the group that received postoperative IV paracetamol.

Kharouba *et al*. (20) recruited 60 patients undergoing dental procedures and allocated them to one of two groups, one in which IV acetaminophen was administered before the procedure and another in which it was administered after the procedure. Pain intensity (using the VAS with faces) and the percentage of patients requiring postoperative analgesia were then compared. The group that received preoperative IV acetaminophen demonstrated a significant reduction in the intensity of postoperative pain, fewer patients requiring pain relief, and lower consumption of postoperative opioids.

Khalili *et al*. (21) recruited 75 patients undergoing lower extremity surgery and allocated them to one of three groups, one that received IV acetaminophen half an hour after the operation, one that received IV acetaminophen prior to skin closure, and one that received normal saline as a placebo. Pain intensity (using a verbal rating scale) and total rescue analgesic consumption were then compared. Pain scores were lower in the preemptive and postoperative groups 6 hours after surgery than in the placebo group. There were no pain score differences after 6 hours among the three groups. Total analgesic consumption 24 hours after surgery was lowest in the preemptive acetaminophen group.

Although there is evidence that the serotonergic pain pathway is acted on by acetaminophen, the full mechanism of action is unclear. It is understood that acetaminophen is very efficient after IV administration, primarily because of its ability to cross the blood–brain barrier (21). Interference in the serotonergic pathways and inhibition of serotonin synthesis by P-chlorophenylalanine both significantly reduce the analgesic effect of acetaminophen (21). In addition, acetaminophen weakly inhibits prostaglandin synthesis *in vitro* and appears to have very little anti-inflammatory activity, although some reduction of tissue swelling after dental surgery has been reported (22).

One of the major advantages of acetaminophen is the low frequency of side effects compared with other non-opioid analgesics used for the treatment of postoperative pain (23). Systematic reviews (24) have found that the rate of adverse events following the administration of paracetamol is not significantly different than that following the administration of a placebo, and hypersensitivity reactions are rare. NSAIDs are commonly used in the postoperative setting but are associated with multiple adverse effects, including gastrointestinal bleeding, cardiovascular effects, and NSAID-induced nephrotoxicity (25). Furthermore, the adverse effects of NSAIDs on mucosal integrity and platelet function are associated with an increased risk of bleeding, a complication that can be particularly problematic in the postoperative setting (26).

A major limitation of this study is the short survey period after surgery. Efficacy should therefore be tested using a longer survey period. Another limitation of this study is the lack of objective assessment of pain relief. We did not find differences in pain relief, as measured by the self-reported VAS, among the three groups despite the patients knowing how to fill the report. If different assessments, such as the face rating scale or verbal rating scale, were used at the same time, a different result may have been attained.

In conclusion, we examined the effect of preemptive acetaminophen on postoperative pain and rescue analgesic use in patients who underwent lower third molar surgery. There was no significant difference in postoperative pain intensity among the three treatment groups. However, the total consumption of rescue analgesics was significantly lower and the time until initial rescue analgesic use was longer in the preemptive IV acetaminophen group. This study makes a novel contribution to the literature by proposing the role of preoperative IV acetaminophen in pain management following third molar surgery under sedation.
